# Approaches to demonstrating the effectiveness of filovirus vaccines: Lessons from Ebola and COVID-19

**DOI:** 10.3389/fimmu.2023.1109486

**Published:** 2023-02-02

**Authors:** Marion F. Gruber, Steven Rubin, Philip R. Krause

**Affiliations:** ^1^ International AIDS Vaccine Initiative, New York, NY, United States; ^2^ GlaxoSmithKline (GSk), Rockville, MD, United States; ^3^ Independent Consultant, Bethesda, MD, United States

**Keywords:** filovirus, Ebola, Marburg, Sudan, vaccine, effectiveness, correlates of protection, immunobridging

## Abstract

Zaire ebolavirus (EBOV), Sudan ebolavirus (SUDV) and Marburg virus (MARV), are members of the *Filoviridae* family that can cause severe disease and death in humans and animals. The reemergence of Ebola, Sudan and Marburg virus disease highlight the need for continued availability of safe and effectives vaccines as well as development of new vaccines. While randomized controlled trials using disease endpoints provide the most robust assessment of vaccine effectiveness, challenges to this approach include the unpredictable size, location, occurrence and duration of filovirus disease outbreaks. Thus, other approaches to demonstrating vaccine effectiveness have been considered. These approaches are discussed using examples of preventive vaccines against other infectious diseases. In addition, this article proposes a clinical immunobridging strategy using licensed EBOV vaccines as comparators for demonstrating the effectiveness of filovirus vaccine candidates that are based on the same licensed vaccine platform technology.

## Introduction

Viral haemorrhagic fever is a deadly disease in humans and nonhuman primates (NHPs) caused by two genera of the larger virus family of *Filoviridae*. The most commonly known belong to the genera Ebolavirus and Marburgvirus. Of the six known Ebolavirus species, four can cause Ebolavirus disease (EVD) in humans: Zaire Ebolavirus (EBOV), Sudan ebolavirus (SUDV), Tai Forest virus (TAFV) and Bundibugyo virus (BDBV) (1). Marburg Virus disease (MVD) usually appears in sporadic outbreaks throughout Africa and is caused by the Marburg virus (MARV) which is a genetically unique virus of the filovirus family (2). The members of the *Filoviridae* family share a common mechanism of action with regard to tropism, cellular and disease pathology (3–6). Also, the genomic organization of Ebolaviruses and Marburgvirus is highly similar with seven sequentially arranged genes encoding: the nucleoprotein (NP), the virion protein 35 (VP35), the VP40, the glycoprotein (GP), the VP30, the VP24, and the polymerase (L). The surface of the filovirus virion is coated by spike-like projections of the GP, which is responsible for the viral antigenicity upon entry and is the target of virus neutralizing antibody (7). Although there is no established immune correlate of protection for filoviruses, levels of GP-binding antibody have been linked to protection (8, 9).

Historically, EBOV has caused most filovirus disease outbreaks and cases. The EBOV outbreak of 2014 - 2016 in West Africa caused over 28,000 cases of EVD and more than 11,000 deaths and led to the rapid development of preventive vaccines against EVD (10). In response to the reemergence of large outbreaks of this deadly disease, several EBOV vaccines were rapidly developed. ERVEBO, a live attenuated, replication competent recombinant vesicular stomatitis virus (rVSVΔG-ZEBOV-GP) vaccine expressing the GP antigen of EBOV was licensed in 2019 by the US Food and Drug Administration (FDA) and the European Medicines Agency (EMA) (11, 12). Effectiveness was demonstrated in a Phase 3 cluster-randomized ring vaccination study conducted in affected areas during the 2014 - 2016 outbreak (13). Zabdeno/Mvabea (Ad26-ZEBOV/MVA-BN-Filo), a heterologous prime-boost vaccine consisting of the non-replicating adenovirus serotype 26 expressing the EBOV GP and the Modified Vaccinia Ankara (MVA) encoding glycoproteins from EBOV, SUDV, MARV as well as TAFV nucleoprotein, was licensed by EMA in 2020 under the exceptional circumstances pathway (14). Effectiveness of the vaccine was inferred from challenge/protection studies in NHPs and clinical immunogenicity data. Although the booster dose of this vaccine expresses SUDV, MARV, and TAFV antigens in addition to EBOV, the vaccine is only approved for prevention of disease caused by EBOV. An adenovirus serotype 5 (Ad-5 EBOV) vaccine expressing the EBOV GP was licensed by the Chinese Food and Drug Administration, and a heterologous prime boost vaccine consisting of recombinant VSV and Ad-5 expressing EBOV GP was licensed by the Ministry of Health of the Russian Federation, both for emergency use (15). There is currently no licensed vaccine indicated for the prevention of disease caused by SUDV or MARV.

**Figure 1:**
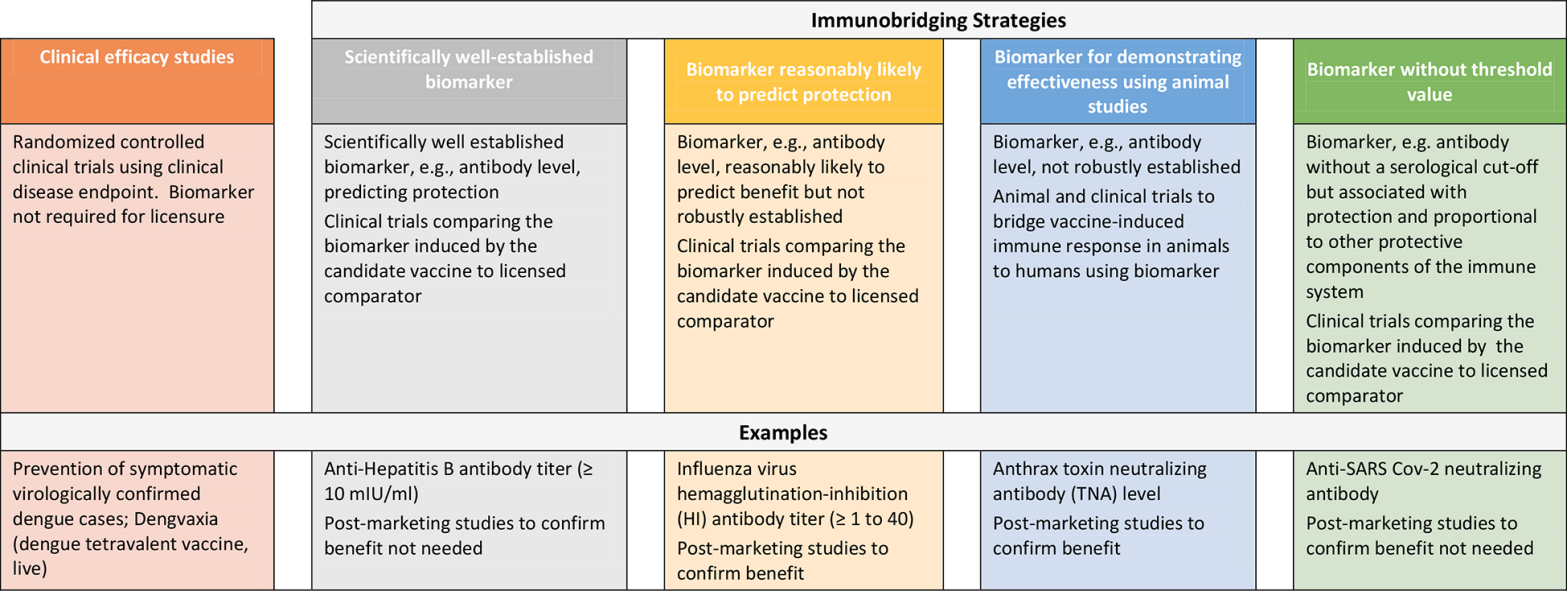
Approaches to demonstrating the effectiveness of preventive vaccines.

Repeated outbreaks of EVD such as the one ending in 2020 in the Democratic Republic of Congo (DRC) and reported cases of MARV in Ghana as well as the SUDV outbreak in Uganda in 2022 underscore the need for additional safe and effective vaccines to protect against filovirus disease (16, 17). However, the sporadic nature of these outbreaks, uncertainties in occurrence and duration and geographic location presents challenges to conducting randomized controlled efficacy trials in particular in preventive settings and thus, other approaches to demonstrating vaccine effectiveness are considered to enable licensure of these products. While approaches to establishing vaccine safety to support licensure are well-established, here we describe strategies to demonstrating vaccine effectiveness using examples of licensed preventive vaccines and present considerations for use of clinical immunobridging strategies to support science-based predictions about the effectiveness of new filovirus vaccine candidates (**Figure 1**).

## Approaches to demonstration of effectiveness of preventive vaccines

### Clinical disease endpoint efficacy studies and/or use of scientifically well-established marker

Randomized controlled clinical trials using prevention of disease as an endpoint represent the gold standard to demonstrate the efficacy of preventive vaccines. Recent examples include DENGVAXIA for the prevention of dengue disease (18) and COMIRNATY and SPIKEVAX for the prevention of disease caused by severe acute respiratory syndrome coronavirus 2 (SARS-CoV-2) (19, 20). For these products, efficacy was demonstrated in pivotal Phase 3 studies using symptomatic virologically confirmed disease as endpoint. Vaccine effectiveness can also be demonstrated using a scientifically well-established and validated marker, e.g, an immune marker, that predicts protection. Examples of such a marker with a defined and validated threshold include anti-Hepatitis B antibody titer (10mIU/ml). This marker has been used as predictors of vaccine effectiveness and has supported licensure of vaccines against Hepatitis B (21). Using this example, in cases where new Hepatitis B vaccines are being developed, in addition to demonstrating attainment of the validated marker of protection, immunological non-inferiority was also demonstrated against the approved comparator product. This is usually based on demonstrating similar geometric mean antibody titers and/or seroconversion rates based on pre-specified statistical criteria.

### Use of immune markers likely to predict protection from disease

Vaccine effectiveness to support licensure has also been inferred based on a surrogate endpoint (e.g, immune marker) thought to be *reasonably likely* to predict clinical benefit even though not robustly established. As in the above section, adequate well-controlled trials comparing the surrogate endpoint in persons administered the candidate vaccine versus the licensed comparator using pre-specified statistical criteria must be conducted. However, as there is uncertainty in regard to ability of the surrogate endpoint to predict effectiveness, post-licensure studies are required to confirm the clinical benefit of the vaccine. In the US, this is referred to as accelerated approval (AA) under 21 CFR 601.40/41 (22). An example of a surrogate endpoints supporting AA include influenza virus hemagglutination-inhibition antibody titer of ≥ 1 to 40 (23). In 2019, FDA convened its Vaccines and Related Biological Advisory Committee (VRBPAC) to discuss data necessary to establish an immunologic marker *reasonably likely* to predict clinical benefit for Chikungunya virus (CHIKV) vaccines (24). Disease outbreaks caused by Chikungunya, like those caused by filoviruses, are irregular and unpredictable making clinical disease endpoint efficacy studies challenging. Furthermore, there is no relevant animal model reflecting clinical CHIKV disease in humans. Data derived from animal models and human epidemiological studies have suggested that CHIKV neutralizing antibody could be used as a surrogate endpoint to support vaccine licensure. Based on these data, FDA and VRBPAC agreed that a CHIKV neutralizing antibody titer *reasonably likely* to predict protection could be established from passive transfer of human antibodies in NHP followed by challenge with wild-type CHIKV. A similar approach could be considered for developing vaccines against SUDV and MARV disease.

### Animal challenge/protection studies

If demonstration of effectiveness is not possible based on a clinical disease endpoint efficacy study, and if a scientifically well-established marker and/or a surrogate endpoint *reasonably likely* to predict protection is not identified, it may be necessary to conduct challenge/protection studies in qualified animal models to demonstrate the effectiveness of the candidate vaccine in preventing disease. Some national regulatory authorities have provisions to allow licensure of a vaccine candidate using this approach (25–27). In the US, this pathway is referred to as the “animal rule” (AR) under 21 CFR 601.91 (28). Under the AR there are specific criteria that must be met including that the animal study endpoint is clearly related to the desired benefit in humans, which is generally the enhancement of survival or prevention of major morbidity. Predicting effectiveness using animal/challenge protection studies includes a) determining that the marker being measured in the animal (usually antibody levels) is associated with protection against virus challenge, b) evaluating the marker in humans and using the information accrued, c) bridging of animal and human data on this marker to establish an effective dose in humans. The vaccine dose in humans should elicit levels of the marker comparable to that of animals protected by the vaccine whereby the dose chosen may not be the same. Furthermore, the marker selected for bridging does not need to be causally responsible for protection.

One example of a vaccine approved based on effectiveness data in animals is BioThrax, anthrax vaccine absorbed (AVA) for post-exposure prophylaxis (PEP) (29). Two General Use Prophylaxis (GUP) challenge/protection studies in rabbits and NHPs studies were performed to estimate protective antibody levels measured in a validated anthrax toxin neutralizing antibody (TNA) assay. TNA levels corresponding to 70% survival probability in the animals were determined. Immunogenicity data in animals were then bridged to human immunogenicity data. The proportion of clinical study subjects achieving a TNA response corresponding to 70% survival probability in animals was determined to estimate effectiveness of the vaccine in humans.

Another example, although not U.S. approved under the AR, is the Zabdeno/Mvabea, Ad26.ZEBOV/MVA-BN-Filo EBOV vaccine for which marketing authorization by EMA in 2020 was based on data demonstrating that immunization with this prime-boost vaccine fully protected NHPs against a lethal EBOV exposure (14). Data on immunogenicity and survival outcome were derived from NHP challenge/protection studies using the selected vaccine dose regimen and a 56-day dose interval. To infer effectiveness of the vaccine in humans, immunobridging was performed based on EBOV GP-binding antibodies measured by the validated EBOV GP FANG ELISA assay used for quantitation of both human and NHP anti-GP IgG. A similar approach could be considered for developing vaccines against SUDV and MARV disease; however, differences between the immune responses in NHPs and humans vaccinated with EBOV vaccines raise some uncertainty with this approach to identifying levels of antibody that would predict protection (30).

### Inference of effectiveness using clinical immunobridging studies

Clinical immunobridging refers to studies in which the effectiveness of a new vaccine candidate is inferred by comparing the vaccine-induced immune response (e.g, neutralizing antibody titer) to that induced by a comparator vaccine for which efficacy was previously demonstrated. The above include examples where clinical immunobridging studies were conducted to infer effectiveness of the candidate vaccine using either a scientifically well-established immune marker or a marker *reasonably likely* to predict protection at a defined threshold. However, immunobridging can also be used as an important tool in the absence of an agreed upon serological cut-off or threshold value of a selected immune marker. Using this approach, one key consideration is that the immune response measured (e.g, neutralizing antibody, total binding antibody) is correlated to protection against disease and is also positively correlated with other protective components of the immune response. In addition, the efficacy of the comparator vaccine will inform statistically appropriate criteria (non-inferiority vs. superiority).

Clinical immunobridging studies have been conducted to demonstrate the effectiveness of COVID-19 vaccines against COVID-19 variant of concerns (VOCs) and new COVID-19 vaccines using neutralizing antibody titers as biomarkers (31). Note that in these cases a correlation between neutralizing antibodies and protection has been confirmed across different vaccine modalities or platforms even though an antibody threshold has not been established (32). In the US, these recommendations pertain to modified vaccines generated using the same process and manufacturer as the authorized or approved parental or “prototype” vaccine. Other regulatory authorities, including the Public Health Agency of Canada (PHAC) have accepted immunobridging studies to authorize not only modified versions of COVID prototype vaccines, but also new COVID vaccines (e.g, vaccines produced by a different manufacturing process) despite the lack of an established correlate or surrogate marker of protection (33). WHO has also promulgated a framework for immunobridging of COVID vaccine efficacy, focusing on the ability of viral neutralizing antibody responses to predict other immune mechanisms of protection for any given vaccine, as well as the effectiveness of the comparator (34).

Similar to COVID-19, although there is no established level of SUDV or MARV GP-specific antibody responses predicting protection against EVD or MVD, levels of filovirus GP-binding antibody are associated with protection against disease (8, 9). Thus, using the analogy of COVID-19, clinical immunobridging studies using a licensed comparator vaccine based on the same platform could be considered for filoviruses. For example, the EBOV GP insert in the licensed rVSVΔG-ZEBOV-GP vaccine, ERVEBO, could be replaced by the SUDV or MARV GP, followed by an immunobridging study demonstrating that the level of anti-GP antibody induced in subjects is comparable to that induced by the parental prototype vaccine. This approach would require confidence that anti-GP antibody responses could predict protection at similar levels for different filoviruses. This confidence is enhanced if the efficacy of the original vaccine is high (as indeed, it is for rVSVΔG-ZEBOV-GP vaccine) and if the immunopathogenesis of the diseases are similar, including rates of disease evolution and potential immune evasion mechanisms used by each virus.

## Discussion

Some or all of the approaches to demonstrating vaccine effectiveness described in this article may be considered to demonstrate the effectiveness of new filovirus vaccine candidates recognizing that each approach presents with challenges and uncertainties. As stated, the sporadic nature of Filoviral outbreaks may not allow the demonstration of protection against EVD and MVD by way of conducting clinical disease endpoint efficacy studies unless there is timely availability of filovirus vaccine candidates at the time of a large outbreak as was the case during the EBOV outbreak in West Africa in 2014 - 2016. Furthermore, there is no scientifically well-established validated immunologic marker that predicts protection against EVD or MVD disease.

There are some important considerations for demonstrating effectiveness based on a surrogate endpoint *reasonably likely* to predict protection for MARV, EBOV or SUDV vaccines. Notably, one must identify a surrogate endpoint, e.g., neutralizing antibody, binding antibodies or cellular immune markers, *reasonably likely* to predict protection. These immune markers may be derived from naturally infected or exposed and protected humans including those participating in vaccine clinical trials in outbreak areas. They may also be derived from animal challenge protection studies (e.g, NHPs). Furthermore, when evaluating whether a particular immune marker is *reasonably likely* to predict protection against EVD or MVD, the conclusion may be different for vaccine candidates that are based on different platforms. For example, immune responses induced by differing vaccine modalities (e.g, a replication deficient-, a replication-competent-, or inactivated virus, a recombinant protein-based and/or nucleic acid-based product) will likely be different, not only in magnitude, but also in the type and breadth of the immune mediators induced (35). It may also be different for virus species that are either homologous or heterologous to the vaccine targeting antigens. Finally, there is a requirement that the immunologic assays used to demonstrate effectiveness are validated.

Demonstration of filovirus vaccine effectiveness using challenge protection studies in animals can be considered if it cannot be demonstrated by other approaches. For filoviruses, the NHP represents an adequate animal model and the disease presentation between humans and NHPs is similar (36–40). However, comparing disease courses between experimentally infected NHPs and naturally infected humans is difficult as route of exposure and challenge dose selected may not resemble natural exposure. Furthermore, there is currently no established EBOV GP antibody titer threshold value associated with clinical benefit. Moreover, studies have demonstrated that immune responses in animals vaccinated with EBOV vaccines are higher than those induced in humans, resulting in uncertainties regarding level of antibody that would predict protection (41).

Numerous studies have been conducted to characterize both vaccine-induced and naturally acquired immunity to filoviruses in humans and animal models. Data indicate that both humoral and cell-mediated immune responses are critical in protecting from filovirus disease (42–44). In NHPs, although cell-mediated immunity plays a role in protection from disease, vaccine effectiveness was consistently associated with the presence of ELISA IgG (45–48). Monoclonal antibodies isolated from human survivors of EVD in the 2014 - 2016 outbreak in West Africa afforded protection in animal EBOV challenge model (49, 50). Human monoclonal antibodies were licensed by FDA for the treatment of infections caused by EBOV in adult and pediatric populations (51, 52). Grais et al. assessed antibody levels induced by the licensed rVSVΔG-ZEBOV-GP vaccine, ERVEBO, using serology data from participants of three immunogenicity trials conducted in Guinea, Sierra Leone and Liberia during the time of the EBOV outbreak in 2014 – 2016 (41). Their analysis supported the Ebola GP-ELISA as a tool for predicting vaccine effectiveness even though contributing protective effects afforded by cell-mediated immunity could not be excluded. However, it is likely that all filovirus vaccines using the rVSVΔG platform will induce cellular responses in similar proportion to humoral responses, supporting use of humoral responses to predict overall responses including cellular responses.

Together, even though the underlying immune mechanism affording protection against filovirus disease is not fully elucidated, anti-GP antibodies play a significant role in providing protection against EVD and MVD. Thus, clinical immunobridging studies using anti-GP ELISA-based IgG levels as an endpoint should be considered to infer effectiveness of new filovirus vaccines. For example, demonstration of statistically pre-specified anti-GP antibody titers induced by the licensed rVSVΔG-ZEBOV-GP vaccine (for which efficacy was demonstrated) and vaccine candidates using the same platform and modified to express the SUDV or MARV GP could potentially serve as the basis for vaccine approval much in the same way as modified COVID-19 vaccines are approved to address VOCs. Importantly, because mechanisms of protection may vary by vaccine platform, such a clinical immunobridging strategy is likely only applicable to vaccine candidates that are based on the same or similar platform as that of the licensed comparator vaccine. This strategy was discussed by global regulators at a recent workshop entitled “ Realizing the potential of correlates of protection for vaccine development and licensure” sponsored by Wellcome held in London, UK, in September 2022. Regulators considered clinical immunobridging studies to infer effectiveness of filovirus vaccine candidates a useful approach provided supportive data would be available. Such data should consist of challenge/protection studies in NHPs demonstrating protective effectiveness of filovirus vaccine candidates against the respective challenge viruses (e.g, SUDV, MARV). Of note, while data derived from challenge/protection studies in animal models would be supportive of the clinical immunobridging strategy, this approach would not be an approval under the AR as the primary data would be derived from comparison of human clinical immunogenicity. Additional supportive data should provide evidence that the pathogenicity and immune mechanism of protection for the filoviruses are similar and that the immune response (humoral and cell mediated immune response) induced by the filovirus candidate vaccines is comparable to that induced by the licensed comparator. The importance of validated assays to assess the immune response induced by the various filovirus vaccine candidates was stressed.

Regardless of the approach chosen to demonstrate effectiveness of filovirus vaccine candidates, clinical safety studies to support a favorable benefit risk ratio of the vaccine will be essential. In addition, real world effectiveness studies of the vaccine post-licensure in the event of an outbreak should be conducted to confirm clinical effectiveness.

In summary, additional vaccines to protect people from filovirus disease in endemic areas, notably Africa, are critically needed. There are a number of approaches to demonstrating vaccine effectiveness including clinical disease endpoint efficacy trials, use of scientifically well-established immune markers, surrogate endpoints *reasonably likely* to predict protection and challenge/protection studies in adequate animal models. In addition, we propose clinical immunobridging studies comparing filovirus vaccine candidates to licensed filovirus comparator vaccines as an approach to infer vaccine effectiveness. Clinical immunobridging has the advantage of being able to directly bridge to clinical efficacy data by way of the licensed comparator vaccine. This approach would need to be supported by data derived from challenge/protection studies in animal models, data on the pathogenesis and protective immune mechanisms for filoviruses and a characterization of the immune response induced by the vaccines. In all cases, the combined data will need to support reasonable likelihood of clinical benefit and a favorable benefit-risk profile. It is the preponderance and strength of the evidence that will determine the licensure pathway used by regulatory authorities.

## Data availability statement

The original contributions presented in the study are included in the article/supplementary material. Further inquiries can be directed to the corresponding author.

## Author contributions

All authors listed have made a substantial, direct, and intellectual contribution to the work and approved it for publication.
